# Chaperone Therapy in Fabry Disease

**DOI:** 10.3390/ijms23031887

**Published:** 2022-02-08

**Authors:** Frank Weidemann, Ana Jovanovic, Ken Herrmann, Irfan Vardarli

**Affiliations:** 1 Department of Medicine I, Klinikum Vest GmbH, Knappschaftskrankenhaus Recklinghausen, Academic Teaching Hospital, Ruhr-University Bochum, 45657 Recklinghausen, Germany; frank.weidemann@klinikum-vest.de; 2 The Mark Holland Metabolic Unit, Nothern Care Alliance NHS Foundation Trust, Salford M6 8HD, UK; Ana.Jovanovic@nca.nhs.uk; 3 Department of Nuclear Medicine, University Hospital Essen, 45147 Essen, Germany; ken.herrmann@uk-essen.de

**Keywords:** Fabry disease, therapy, chaperone

## Abstract

Fabry disease is an X-linked lysosomal multisystem storage disorder induced by a mutation in the alpha-galactosidase A (GLA) gene. Reduced activity or deficiency of alpha-galactosidase A (AGAL) leads to escalating storage of intracellular globotriaosylceramide (GL-3) in numerous organs, including the kidneys, heart and nerve system. The established treatment for 20 years is intravenous enzyme replacement therapy. Lately, oral chaperone therapy was introduced and is a therapeutic alternative in patients with amenable mutations. Early starting of therapy is essential for long-term improvement. This review describes chaperone therapy in Fabry disease.

## 1. Introduction

Fabry disease (FD) is a multisystem lysosomal storage disorder induced by a mutation in the alpha-galactosidase A (GLA) gene located on the X chromosome [1]. Reduced activity or deficiency of alpha-galactosidase A (AGAL) is leading to escalating storage of intracellular globotriaosylceramide (GL-3) in numerous organs, including the nervous system, kidneys and heart [2]. Typical manifestations include peripheral neuropathic pain, gastrointestinal symptoms, angiokeratoma, anhidrosis, left ventricular (LV) hypertrophy, cornea verticillate, renal failure or cryptogenic stroke [3,4,5,6,7,8]. Several articles have been published on the diagnosis and treatment of FD [9,10,11].

In addition, measurement of AGAL activity is highly recommended. In males, reduced AGAL activity (<1% of the mean normal) is extremely suggestive of classic FD [9]. In females and in patients with late-onset mutations, the enzyme activity may be residual or even in a normal range; thus, in such cases, genetic testing for Fabry mutations is essential [12]. The supplementary measurement of globotriaosylsphingosine (Lyso-Gb3) is advocated. Lyso-Gb3 levels ≥2.7 ng/mL are pathological and in general the level of Lyso-Gb3 is related to disease activity and to the severity of mutation [13]. In addition, Lyso-Gb3 can be used to monitor therapy effects during specific treatment such as chaperone therapy. For assessment of important cardiac involvement, measurements of highly sensitive Troponin (hsTnT) and B-type natriuretic peptide (NT-proBNP), as biomarkers, are recommended [14]. Whereas hsTnT indicates mainly myocardial fibrosis in hypertrophic left ventricles, NT-proBNP is elevated in end-stage Fabry cardiomyopathy. Both blood biomarkers are part of the important diagnostic tools to initiate and to monitor chaperone therapy.

Even though blood tests are comfortable to do, a large number of patients are diagnosed late during the disease progression, because symptoms can vary extremely and thus it is challenging to designate broad clinical symptoms to this very rare disease. Overall, it takes 10 years from the first symptom to the appropriate diagnosis of FD.

In patients with FD, morbidity and poor prognosis are largely driven by cardiomyopathy [15,16]. The usual treatment of FD is enzyme replacement therapy (ERT), either Replagal^®^ (Agalsidase alfa) or Fabrazyme^®^ (Agalsidase beta), and since a few years ago chaperone therapy Migalastat [17]. In end-stage cardiomyopathy, patient heart transplantation should be considered [18]. Regardless of the kind of treatment concept, starting the therapy early is key for long-term prognosis [19]. For future therapy options, several therapeutic approaches along with gene therapy are under development. This review describes the chaperone therapy in Fabry disease.

## 2. Chaperone Therapy from Bench to Bedside

Some patients with FD have missense mutations with normal AGAL catalytic activity, but a reduction in overall AGAL enzymatic activity due to reduced stability of the mutated protein, caused by protein misfolding and premature degradation [20,21]. To correct for this misfolding and to prevent premature degradation a small molecule called chaperone was developed.

In 1995, Okumiya et al. proved that galactose stabilized a missense mutation of AGAL. In their in vitro experiment, they demonstrated that after administering 100 mM galactose to cells expressing a missense mutant an increasing amount of AGAL protein could be recorded and a higher activity of the protein was present [22]. Thereafter, Frustaci et al. used a very high dosage of galactose infusion (1 g/kg body weight) in a Fabry patient and showed an improvement of the present cardiomyopathy [23]. However, it was clear that such a high dosage of galactose is not practicable in patients with Fabry disease [23]. Thus, other small molecules were tested in cells with missense mutations for AGAL such as 1,5-dideoxy-1,5-iminogalactitol, which is an iminosugar analog of galactose was one of the promising candidates [24]. The major advantage of this iminosugar was that the biological half-time period was far longer than galactose [24]. This small molecule was chemically called 1-deoxygalactonojirimicin (DGJ). Fan et al. proved in 1999 that DGJ was more effective than galactose in stabilizing responsive AGAL mutants [25]. They proposed a concept of a chemical chaperone as a small molecule which can assist a protein to fold properly allowing it to enter physiological processing pathways smoothly [25]. Later on, the concept was called a pharmacological chaperone and for missense mutations in Fabry disease, it is known as Migalastat. In 2018, this therapy was approved by the FDA for amenable mutations [26] and the trademark name is Galafold^®^ [24].

## 3. Chaperone Therapy Concept

**Figure 1:**
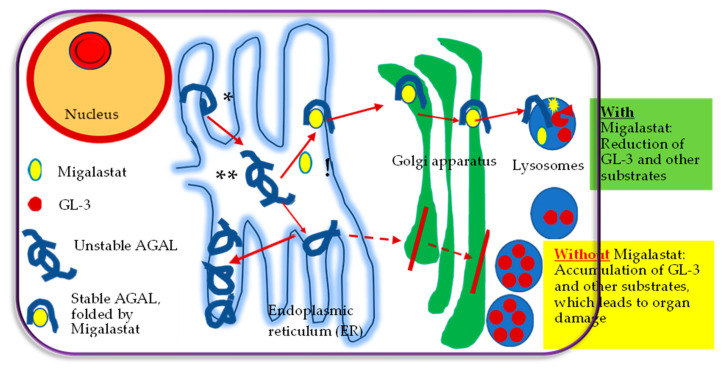
Overview of action of Migalastat. AGAL, alpha-galactosidase A. GL-3, Globotriaosylceramide. (*) Synthesis of misfolded AGAL. (**) Accumulation of misfolded AGAL.

As described above galactose was used in the first in vitro studies. The enzyme activity in vitro was increased after adding galactose to the culture medium of COS-1 cells with the p.Q279E mutation [20]. However, it has been shown that galactose did not increase enzyme activity in AGAL mutations [22]. Later studies mostly used 1-deoxygalactonojirimycin (known as Migalastat, Galafold^®^, Amicus Therapeutics, U.S., Inc., Philadelphia, PA, USA), a galactose analogue, in which oxygen is replaced by a nitrogen atom in the ring. As shown in Figure 1, Migalastat binds to the active site of amenable mutant forms of AGAL. This is stabilizing AGAL and prevents its degradation in the endoplasmic reticulum by properly folding of AGAL. Due to the efficient “quality control” in the endoplasmic reticulum, transport to the Golgi apparatus is limited only to properly folded and assembled proteins. The mutant AGAL is retarded in the normal pathway, leading to deficiency of AGAL activity [25]. Thus, stable AGAL folded by Migalastat migrates from the endoplasmic reticulum to the Golgi apparatus for further processing and transport to the next destination, the lysosomes. Migalastat dissociates in the lysosomes from AGAL, granting the enzyme to apply its activity on GL-3 [27,28,29]. Here, GL-3 is broken down into lactosylceramide by AGAL. In summary, chaperone therapy with Migalastat elevates correct folding of the mutated protein and increases its stability, which leads to a reduction of GL-3 and its substrates (Figure 1).

Migalastat is also a strong competitive inhibitor of AGAL [10]; however, at lower doses, it increases enzymatic activity for amenable AGAL mutations [25], as described in Figure 1.

### 3.1. Migalastat

Currently, Migalastat is the only small molecule oral treatment for FD, administered 123 mg once every other day. Migalastat was approved by the European Medicines Agency (EMA) in 2016, and by the US Food Drug Administration (FDA) in 2018 for the treatment of patients aged ≥12 years, with estimated glomerular filtration rate (eGFR) ≥30 mL/min/1.73 m^2^ and amenable AGAL mutations [30,31,32,33]. Migalastat is not recommended in patients pregnant or breastfeeding [31,32,34]. Due to the rarity of FD, large clinical trials with Migalastat were not performed yet.

Migalastat has been approved in 43 countries worldwide and is available in 33 of them (e.g., Argentina, Colombia and Brazil); the combined market experience worldwide on top of studies is over 3500 patient years.

Compared to ERT, Migalastat therapy has considerable advantages: (a) it is non-immunogenic, escaping antibody-related tolerability; (b) it is an oral treatment; (c) it may lead to sustained enzyme levels that more closely mimic the endogenous enzyme(d) potentially improved tissue and cellular distribution; (e) and capability to pass the blood–brain barrier [17], as implied by the reporting of enhanced AGAL activity and diminished GL-3 levels in the brain of Fabry transgenic mice [35,36].

Wu et al. assessed Migalastat and agalsidase beta biodistribution in mice. Their results suggested that Migalastat had broad tissue distribution in the heart, kidney and small intestine [37]. They showed that Migalastat might be distributed to tissues with limited access to intravenous agalsidase beta [37].

#### 3.1.1. Amenability to Migalastat

Some mutations and especially missense mutations result in a misfolded AGAL protein which leads to reduced enzyme activity. The misfolded protein cannot enter the lysosomes and thus premature degradation in the endoplasmic reticulum leads to storage of Gb3 [38]. Before starting to treat an FD patient with Migalastat, the amenability of the specific mutation has to be evaluated. Amenability implies the pharmaceutical response with respect to increasing enzyme activity of the AGAL mutation after incubation with Migalastat. Several different in vitro assays have been developed to evaluate amenability [39,40,41]. The basic concept behind the assays is comparable as they all work with cell culture in human embryonic kidney cells (HEK). Lately, the good laboratory practice (GLP) HEK assay by Benjamin et al. seems to be the gold standard and only patients with amenable mutations assessed by this method should be treated with Migalastat [30]. This assay has been clinically evaluated [30]. Some AGAL mutations are classified as non-amenable; such mutations include insertions, truncations, splicing mutations, large mutations and frameshift mutations, and do not pass for testing. Patients with mutations in the GLA gene are believed to be eligible for treatment with Migalastat if the amended AGAL activity rises at least 1.2-fold, with an absolute increase in activity greater than 3% of the enzymatic activity of the wild-type AGAL [30]. The extremely variable biochemical response to the Migalastat therapy may be explained by the extensive range of increase in AGAL activity (1.2–30.4-fold) after treatment with Migalastat [30]. About 35–50% of FD patients have amenable AGAL mutations to Migalastat [32]. Nonetheless, in vitro and in vivo amenability may not always match, as an insufficient increase of the enzymatic activity and rising values of plasma lyso-Gb3 in patients with certain AGAL mutations (classified as amenable on the basis of the in vitro GLP-HEK assay) have been shown in recent trials [38,42,43]. Consequently, surveillance of the clinical response and consecutive measurements of the AGAL enzymatic activity in leukocytes and lyso-Gb3 are essential for assessing in vivo amenability to Migalastat [36]. The website (https://www.galafoldamenabilitytable.com/hcp, accessed on 4 November 2021) provides information on the amenability of AGAL mutations to Migalastat [44].

By using the in vitro information about amenability and as well as data on the clinical response (in vivo amenability) four groups of amenable mutations should be considered [38]: (1) Patients with amenable mutations (e.g., N215S) with typical Fabry symptoms which benefit clinically very well from Migalastat therapy, (2) Non-pathogenic amenable mutations of genetic variants. These patients should not be treated with Migalastat or other disease modifying therapy. (3) Amenable mutations that do not respond to Migalastat. In these patients, alternative therapy concepts should be considered. (4) It is conceivable that in a few mutations Migalastat acts as an inhibitor with a very strong affinity to the center. Consequently, a potential increase in lyso-Gb3 could be observed. If these patients clinically do not respond, Migalastat should be discontinued [38].

#### 3.1.2. Efficacy of Migalastat

Migalastat therapy is applicable to ERT-naïve as well as to ERT-experienced patients. Switching from ERT to Migalastat is also possible.

There are two relevant phase 3 trials on chaperone therapy in FD patients, the FACETS trial and ATTRACT trial.

**Figure 2:**
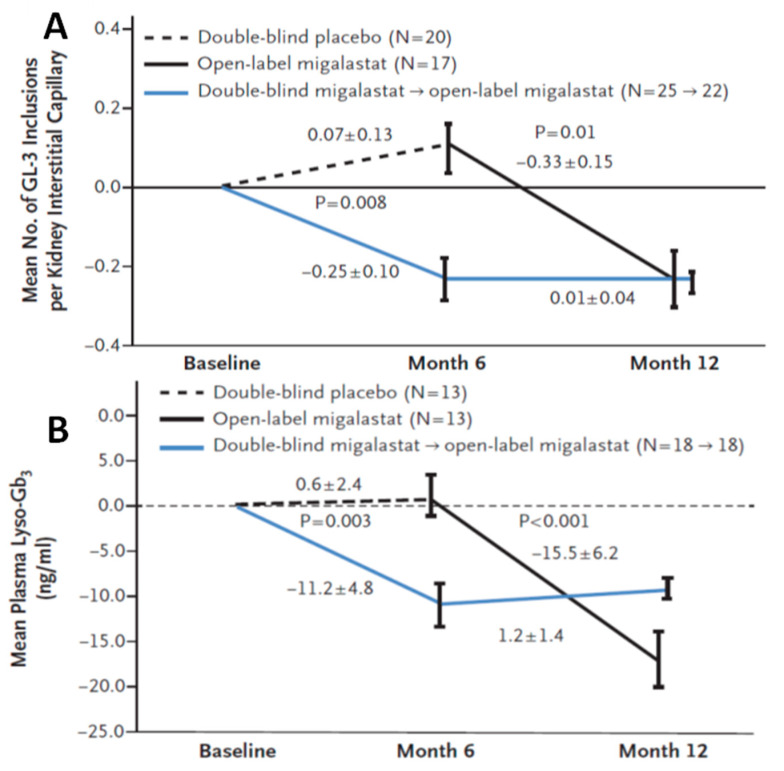
(**A**) Difference from baseline in kidney interstitial capillary globotriaosylceramide (GL-3) in patients with mutant α-galactosidase forms that were suitable for Migalastat treatment [31]. An analysis of covariance (ANCOVA) model with covariate adjustment for baseline value and factors for therapy group and therapy-by-baseline interaction was used for the difference from baseline to month six, the *p* value of 0.008 corresponds to the least-squares mean differences between Migalastat and placebo. A mixed effects model for repeated measures was used for the difference from month 6 to month 12 in patients switching from placebo to Migalastat. The model used fixed effects for therapy group and time, time-by-therapy interaction, and baseline GL-3 inclusion. I bars indicate standard errors (SEM) [31]. (**B**) Change from baseline in plasma globotriaosylsphingosine (lyso-Gb3) levels in patients with suitable mutant α-galactosidase. Baseline values were normalized to zero, and data represent the mean difference from baseline of month six. An ANCOVA model was used to compare Migalastat with placebo from baseline to month six and to compare the difference from six to month 12 in patients changing from placebo to Migalastat. The ANCOVA model used covariate adjustment for baseline value and factors for therapy group and therapy-by-baseline interaction. *p* values correspond to the least-squares mean difference between Migalastat and placebo. Of the 44 patients who allowed plasma lyso-Gb3 analyses, 31 had suitable mutant α-galactosidase. I bars indicate standard errors (SEM) [31]. (**A**,**B**) adapted from Germain et al., with permission [31].

In the FACETS trial, 67 patients were randomized to six months double-blind Migalastat or placebo, followed by open-labeled Migalastat for six to 12 months. During the trial, patients were monitored by blood biomarkers, Fabry symptoms evaluation, clinical assessment for kidney function and heart morphology and by histology assessment with kidney biopsies. In the modified-intention to treat group (ERT-naïve patients with Migalastat-amenable AGAL mutations), plasma lyso-Gb3 (as a blood disease biomarkers) and the mean number of GL-3 inclusion in renal biopsy (for histology assessment) were significantly reduced by Migalastat at six months (Figure 2).

**Figure 3:**
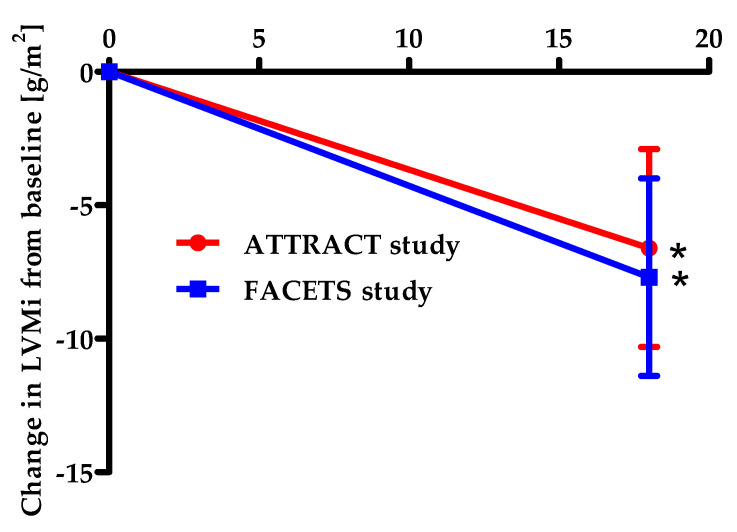
Differences in echocardiographic-derived Left-ventricular-mass (LVM) index change from baseline to at least 18 months on Migalastat [31,32]. **Red line:** In the ATTRACT study, difference to month 18 in mITT patients (all randomized, treated patients with amenable mutations). LVMi decreased significantly (95%CI; 6.6 (−11.0 to −2.2 *) in patients switched from enzyme replacement therapy (ERT) to Migalastat [32]. **Blue line:** in the FACETS study. Patients in the intention-to-treat population who had suitable mutant α-galactosidase, underwent echocardiography baseline and post-baseline, and received Migalastat for at least 18 months†. Month six was used as the baseline for patients who received placebo for 6 months before switching to Migalastat. LVMi decreased significantly (95%CI; −7.7 (−15.4 to −0.01 *) [31]. Values are means ± SEM, * The difference from baseline was considered to be significant because the 95% confidence interval did not include zero, †Month 18 or 24 was used as the baseline of the extension study, LVMi, left ventricular mass index, mITT, modified intention-to-treat population, CI, confidence interval.

In addition, the mean total GL-3 inclusion volume per podocyte in renal biopsies decreased by the Migalastat therapy from baseline to 6 months [31,45]. This was an important finding as clearance of podocytes from GL-3 is very difficult. For kidney function, eGFR, measured glomerular filtration rate (mGFR), 24 h urine protein excretion, and 24 h urinary GL-3 showed no significant differences between the groups at baseline levels and insignificant variations from baseline to month 6 [31,45]. In contrast, there was a notable reduction of the mean LV mass index (for heart morphology) relative to the baseline at 18 or 24 months of Migalastat therapy (Figure 3) [31,32].

For assessment of typical Fabry symptoms 6 months after treatment with Migalastat, an improvement in diarrhea was found [31,45,46]. In the subgroup of classic males from the FACETS trial those results could be confirmed, regardless of disease severity [47].

In the 18-month, randomized, active-controlled ATTRACT trial, 57 ERT-experienced patients, 53 of them with amenable AGAL mutations were selected to switch to Migalastat or to continue ERT. Migalastat and ERT had comparable effects on renal function. Plasma lyso-Gb3 levels stayed low and stable after switching from ERT to Migalastat. The mean LV mass index was significantly reduced at 18 months after treatment with Migalastat. The improvement of the LV mass index correlated with variations in the interventricular septum thickness. In contrast, very little change of the mean LV mass index was observed in the ERT group [32]. Furthermore, the open-label extension study done by Feldt-Rasmussen et al. showed a notable reduction of the LV mass index, well tolerability of Migalastat and long-term stability of renal function after 30 months of Migalastat 150 mg every other day in patients with Fabry disease and amenable AGAL variants with LV hypertrophy (LVH) at baseline [48].

In summary, the two randomized trials FACETS and ATTRACT documented a significant reduction of LV hypertrophy (−7.7 g/m^2^ and −6.6 g/m^2^, respectively) after 24 and 18 months of treatment with Migalastat [31,32,49]. In addition, kidney function was stabilized and diarrhea as a clinical symptom was improved during Migalastat therapy. Very importantly, plasma lyso-Gb3 as a parameter for diseases activity decreased in previously untreated patients and remained stable in ERT pretreated patients [31,32,49].

A post hoc analysis of changes in renal function in patients with amenable AGAL variants who were treated with Migalastat for more than 2 years in the phase 3 FACETS and ATTRACT trials, and the long-term, open-label extension (OLE) studies, showed maintained renal function irrespective of the treatment status, gender, or phenotype [50].

Müntze et al. showed that the LV mass index in 14 patients treated with Migalastat for 1 year significantly decreased, while the plasma lyso-Gb3 exhibited a similar trend in naïve patients and was maintained in patients who switched from ERT. They reported significantly reduced eGFR, in contrast to the findings in the pivotal clinical trials, and supposed that this might be explained by the coincident beginning of angiotensin converting enzyme inhibitors. Thus, they recommend further studies with Migalastat with longer follow-up for kidney function [51].

Lenders et al. showed in a prospective observational multicenter study (FAMOUS) including 59 patients that treatment of previously ERT-treated and untreated FD patients with Migalastat for 12 months was safe with a considerable reduction of LV mass index [42]. In patients with systolic blood pressure below 120 mmHg, a higher decline of eGFR was shown. The latter was also reported in females with AGAL mutations and categorized as non-amenable by the in-house assay based on AGAL-knockout HEK cells [42].

A study by Riccio et al. showed a significant decrease of LV mass with seven FD males, who switched from ERT to Migalastat. This was also detected after 1 year of therapy with Migalastat, whereas plasma lyso-Gb3 was not changed significantly. In contrast to other real-life studies, eGFR remained unchanged and proteinuria notably decreased [52].

Headaches and nasopharyngitis have been reported as the most common side effects of generally well-tolerated Migalastat [31,32].

Another clinical disadvantage of Migalastat compared to ERT is that so far only a few trials on Migalastat are available. In addition, experience with ERT during daily clinical work with Fabry patients is much better than with Migalastat. 

## 4. Clinical Workup during Chaperone Therapy

Whenever a new Fabry patient is diagnosed the first step is to classify the AGAL mutation for amenability. For most of the mutations, this can be easily done by checking the mutation of interest in the reference website (https://www.galafoldamenabilitytable.com/hcp, accessed on 4 November 2021). Only patients in which their mutation is assigned as amenable on this website are eligible to be treated with Migalastat. For a better understanding of the clinical impact of the found AGAL mutation the enzyme activity and lyso-Gb3 should be measured additionally.

The next very important step is that an interdisciplinary, collaborative team of Fabry specialists have to evaluate the different organ systems [53]. Comprehensive cardiologic, nephrologic, and neurologic work-ups have to be organized for more life-threatening scenarios (e.g., cardiomyopathy, renal insufficiency, stroke) [53]. In general, Fabry-related specialists have two main duties: (1) to confirm or rule out Fabry disease involvement of the relevant organ system and assess the extent of disease progression; (2) to determine the need for a specific therapy [54].

In patients where the start of a specific therapy is decided the team has to choose either enzyme replacement therapy (ERT) or in amenable mutations chaperone therapy with Migalastat. If Migalastat is chosen every 6 months a clinical follow-up should be performed [38,55]. During every follow-up, the therapeutic goals should be checked [55]. This check should include information on patients’ reported symptoms, inclusive information about quality of life, blood biomarkers inclusive lyso-Gb3 and AGAL activity and a comprehensive evaluation of the different organ involvements. During therapy with Migalastat, the therapeutic goals are: (1) stabilization of organ function, i.e., kidney insufficiency, (2) an improvement of organ function, i.e., decrease in left ventricular mass in patients with a hypertrophic cardiomyopathy, (3) reduction in Fabry related symptoms, (4) improvement of quality of life and (5) decrease of lyso-Gb3 in parallel to an increase of AGAL activity [12,55]. Using all this information, it has to be decided if the therapeutic goals are achieved and thus the patient is clinically amenable. [38]. However, if a patient is clinically non-amenable, before considering discontinuation of therapy, the first step is to check patients adherence [38] and in addition if adjunctive therapy is required [38]. If adherence is ensured the interdisciplinary Fabry specialist should again evaluate the in vitro amenability of the specific mutation by integrating the literature about this mutation and the related amenability [38]. Taking all this information together, in some patients, Migalastat has to be stopped and a switch to ERT should be discussed. In very rare patients a co-medication of ERT together with Migalastat should be considered by the interdisciplinary Fabry team. However, so far, clinical data on this specific therapeutic concept are not available.

## 5. Conclusions

Oral chaperone therapy with Migalastat in patients with Fabry disease is innovative and very safe without severe side effects. Clinical data proved that during therapy with Migalastat LV hypertrophy decreased and kidney function was stabilized in most patients. However, only patients with amenable AGAL mutations to Migalastat are potential candidates for this treatment concept.

## Data Availability

Not applicable.
